# Supplementary material for: Recurrent HBV Integration Targets as Potential Drivers in Hepatocellular Carcinoma
Source: Cells. 2021 May 23;10(6):1294. doi: 10.3390/cells10061294 (PMC8224658; doi:10.3390/cells10061294)
Supplement: Supplementary file 1 [file cells-10-01294-s001.zip › cells-1212399-supplementary.pdf]

Supplemental Table S1. Characterization of HBV-JSs identified in in-house HBV-HCC tissue.

Patient ID	Total NGS Reads	On-target HBV Read %	HBV-host junction breakpoint nucleotide (nt.) position		Gene
			HBV	Human	
1	6.12E+06	1.1%	1773	Chr5: 1295082	TERT
2	6.24E+06	1.1%	1801	Chr5: 1295123	TERT
3	6.35E+06	1.1%	1801	Chr5: 1299125	TERT
4	3.55E+06	0.8%	1820	Chr19: 29812873	CCNE1
5	5.19E+06	1.2%	1827	Chr8: 64147161	LOC102724623
			1795	Chr9: 45073810	Unknown
6	7.24E+06	1.4%	1801	Chr20: 53437062	LINE2
7	7.62E+06	1.3%	1901	Chr19: 29812390	CCNE1
			1765	Chr6: 17125139	STMND1
8	2.11E+06	1.0%	1712	Chr10: 31192627	LOC101929352
9	2.26E+06	1.0%	1623	Chr9: 16709453	BNC2
10	1.17E+06	1.0%	1756	Chr11: 92048629	LINE1
11	2.64E+06	1.1%	1814	Chr5: 1284093	TERT
			1802	Chr19: 29812598	CCNE1
			1765	Chr4: 116834523	HAVCR1P2
			1781	Chr14: 32527123	AKAP6
12	8.71E+06	1.3%	1826	Chr2: 74945009	LOC105369842
			1722	ChrX: 130398440	RBMX2
13	5.78E+06	1.2%	1800	Chr10: 124355242	OAT
14	3.46E+06	0.9%	1825	Chr16: 29467674	LOC388242
15	1.67E+06	1.2%	1783	Chr5: 1299170	TERT
16	3.73E+06	0.8%	1796	Chr14: 67004392	GPHN
17	4.42E+06	0.8%	1772	Chr19: 35403632	LINC01531
18	1.22E+06	1.0%		N.D.	
19	5.04E+06		1803	Chr1: 39524755	Unknown
		1.2%	1811	Chr14: 95571897	LOC100506999
20	5.37E+06	1.2%	1713	ChrX: 35786804	LTR Element
21	9.00E+06	1.3%		N.D.	
22	3.84E+05	0.04%	1727	Chr14: 103176826	LOC105370685
**Avg. ± SD**	4.36E+06 ± 2.60E+06	1.0% ± 0.3%			

The nucleotide positions of the HBV (NC_003977.1) and human (GRCh38.p2) genome sequences at the HBV-human junction breakpoints. Within 100 kb of the HBV integration site breakpoint, the closest genes were identified by *ChimericSeq* software and listed as defined by NCBI's RefSeq gene database. Integration sites where no known gene was present within 100 kb are listed as "Unknown". N.D., no detectable HBV-host junctions; Avg. ± SD, average ± standard deviation.

Supplemental Table S2. Clinical characteristics of in-house HBV-HCC patient cohort (n=22).

Patient ID	Age (years)	Gender (M/F)	Cirrhosis (-/+)	Tumor stage*	Tumor size (cm)
1	71	M	+	1	3.5
2	68	M	-	NA	9.0
3	63	F	+	NA	3.7
4	44	F	-	1	3.5
5	43	M	-	2	3.0
6	68	M	-	1	6.5
7	58	M	-	2	15.0
8	57	M	-	1	4
9	29	M	+	2	7
10	41	M	+	1	2
11	33	F	+	1	2.5
12	57	M	+	1	3
13	73	M	+	4	11.0
14	49	M	+	2	3.4
15	61	M	-	2	2.3
16	75	F	-	1	3.0
17	47	M	-	2	4.5
18	74	F	+	3A	5.5
19	75	M	-	1	1.9
20	55	F	+	1	4.0
21	46	F	+	4	1.5
22	39	F	-	2	10

*denotes HCC tumors were staged using the tumor-node-metastasis (TNM) staging system.

Supplemental Table S3. Pathways identified by Enrichr GO biological processes analysis of the 28 most frequent RTGs.

Pathway description (Gene ontology accession)	Genes
cell cycle G1/S phase transition (GO:0044843)	CCNA2;CCNE1
histone modification (GO:0016570)	CCNA2;PRMT2
positive regulation of amyloid-beta clearance (GO:1900223)	ROCK1
regulation of endothelial cell development (GO:1901550)	ROCK1
negative regulation of membrane protein ectodomain proteolysis (GO:0051045)	ROCK1
chromatin-mediated maintenance of transcription (GO:0048096)	KMT2B
regulation of myosin-light-chain-phosphatase activity (GO:0035507)	ROCK1
integrin activation (GO:0033622)	FN1
protein desumoylation (GO:0016926)	SENP5
peptidyl-arginine methylation, to asymmetrical-dimethyl arginine (GO:0019919)	PRMT2
regulation of amyloid precursor protein catabolic process (GO:1902991)	ROCK1
peptidyl-arginine omega-N-methylation (GO:0035247)	PRMT2
peptidyl-arginine methylation (GO:0018216)	PRMT2
negative regulation of amyloid-beta formation (GO:1902430)	ROCK1
histone phosphorylation (GO:0016572)	CCNA2
muscle cell migration (GO:0014812)	ROCK1
regulation of amyloid-beta clearance (GO:1900221)	ROCK1
negative regulation of amyloid precursor protein catabolic process (GO:1902992)	ROCK1
histone arginine methylation (GO:0034969)	PRMT2
regulation of actomyosin structure organization (GO:0110020)	ROCK1
regulation of establishment of endothelial barrier (GO:1903140)	ROCK1
smooth muscle contraction (GO:0006939)	ROCK1
regulation of establishment of cell polarity (GO:2000114)	ROCK1
regulation of clathrin-dependent endocytosis (GO:2000369)	ROCK1
regulation of cell-substrate junction assembly (GO:0090109)	ROCK1
positive regulation of gene expression (GO:0010628)	ROCK1;PRMT2;CCNE1;FN1
neuron projection morphogenesis (GO:0048812)	ROCK1;CTNND2
dendritic spine morphogenesis (GO:0060997)	CTNND2
leukocyte tethering or rolling (GO:0050901)	ROCK1
regulation of bicellular tight junction assembly (GO:2000810)	ROCK1
leukocyte adhesion to vascular endothelial cell (GO:0061756)	ROCK1
neuron projection development (GO:0031175)	ROCK1;CTNND2
regulation of establishment or maintenance of cell polarity (GO:0032878)	ROCK1
positive regulation of focal adhesion assembly (GO:0051894)	ROCK1
negative regulation of phosphoprotein phosphatase activity (GO:0032515)	ROCK1
regulation of adherens junction organization (GO:1903391)	ROCK1
regulation of amyloid-beta formation (GO:1902003)	ROCK1
histone methylation (GO:0016571)	PRMT2
negative regulation of cell junction assembly (GO:1901889)	ROCK1
positive regulation of adherens junction organization (GO:1903393)	ROCK1
regulation of epidermal cell differentiation (GO:0045604)	ROCK1
cortical actin cytoskeleton organization (GO:0030866)	ROCK1

regulation of membrane protein ectodomain proteolysis (GO:0051043)	ROCK1
negative regulation of cell cycle G1/S phase transition (GO:1902807)	PRMT2
histone lysine methylation (GO:0034968)	KMT2B
regulation of androgen receptor signaling pathway (GO:0060765)	PRMT2
positive regulation of cell junction assembly (GO:1901890)	ROCK1
histone H3-K4 methylation (GO:0051568)	KMT2B
dendritic spine organization (GO:0097061)	CTNND2
regulation of actin filament bundle assembly (GO:0032231)	ROCK1
negative regulation of G1/S transition of mitotic cell cycle (GO:2000134)	PRMT2
regulation of transcription involved in G1/S transition of mitotic cell cycle (GO:0000083)	CCNE1
GPI anchor metabolic process (GO:0006505)	CWH43
leukocyte cell-cell adhesion (GO:0007159)	ROCK1
regulation of intracellular steroid hormone receptor signaling pathway (GO:0033143)	PRMT2
mitotic cell cycle phase transition (GO:0044772)	CCNA2;CCNE1
bicellular tight junction assembly (GO:0070830)	PARD6G
positive regulation of fibroblast proliferation (GO:0048146)	FN1
Ras protein signal transduction (GO:0007265)	CCNA2;ROCK1
protein alkylation (GO:0008213)	PRMT2
regulation of keratinocyte differentiation (GO:0045616)	ROCK1
cortical cytoskeleton organization (GO:0030865)	ROCK1
dendrite morphogenesis (GO:0048813)	CTNND2
GPI anchor biosynthetic process (GO:0006506)	CWH43
substrate adhesion-dependent cell spreading (GO:0034446)	FN1
endodermal cell differentiation (GO:0035987)	FN1
positive regulation of cell-matrix adhesion (GO:0001954)	ROCK1
apical junction assembly (GO:0043297)	PARD6G
endoderm formation (GO:0001706)	FN1
protein modification by small protein removal (GO:0070646)	CCNA2;SENP5
regulation of cellular amide metabolic process (GO:0034248)	ROCK1
negative regulation of protein catabolic process (GO:0042177)	ROCK1
androgen receptor signaling pathway (GO:0030521)	CCNE1
negative regulation of cytokine secretion (GO:0050710)	FN1
regulation of gene expression (GO:0010468)	ZNF595;ROCK1;PRMT2;FN1
negative regulation of proteolysis (GO:0045861)	ROCK1
positive regulation of macromolecule metabolic process (GO:0010604)	ROCK1;FN1
glycolipid biosynthetic process (GO:0009247)	CWH43
regulation of protein modification process (GO:0031399)	FN1
negative regulation of protein metabolic process (GO:0051248)	ROCK1
regulation of focal adhesion assembly (GO:0051893)	ROCK1
protein methylation (GO:0006479)	PRMT2
regulation of G1/S transition of mitotic cell cycle (GO:2000045)	PRMT2
regulation of cellular component movement (GO:0051270)	ROCK1
regulation of fibroblast proliferation (GO:0048145)	FN1
positive regulation of gene expression, epigenetic (GO:0045815)	KMT2B
peptide cross-linking (GO:0018149)	FN1
regulation of megakaryocyte differentiation (GO:0045652)	KMT2B

positive regulation of cell-substrate adhesion (GO:0010811)	FN1
regulation of cell-matrix adhesion (GO:0001952)	ROCK1
cell morphogenesis involved in differentiation (GO:0000904)	FN1
regulation of cellular catabolic process (GO:0031329)	ROCK1
negative regulation of cellular catabolic process (GO:0031330)	ROCK1
I-kappaB kinase/NF-kappaB signaling (GO:0007249)	ROCK1
regulation of neurogenesis (GO:0050767)	ROCK1
regulation of stress fiber assembly (GO:0051492)	ROCK1
regulation of actin filament-based process (GO:0032970)	ROCK1
negative regulation of blood vessel morphogenesis (GO:2000181)	ROCK1
regulation of myeloid cell differentiation (GO:0045637)	KMT2B
intracellular steroid hormone receptor signaling pathway (GO:0030518)	CCNE1
positive regulation of autophagy (GO:0010508)	ROCK1
negative regulation of protein binding (GO:0032091)	ROCK1
protein sumoylation (GO:0016925)	SENP5
negative regulation of angiogenesis (GO:0016525)	ROCK1
vascular endothelial growth factor receptor signaling pathway (GO:0048010)	ROCK1
Rho protein signal transduction (GO:0007266)	ROCK1
regulation of actin cytoskeleton organization (GO:0032956)	ROCK1
negative regulation of cellular amide metabolic process (GO:0034249)	ROCK1
extracellular matrix disassembly (GO:0022617)	FN1
cell-cell junction assembly (GO:0007043)	PARD6G
negative regulation of mitotic cell cycle phase transition (GO:1901991)	PRMT2
negative regulation of NF-kappaB transcription factor activity (GO:0032088)	PRMT2
negative regulation of binding (GO:0051100)	ROCK1
ephrin receptor signaling pathway (GO:0048013)	ROCK1
regulation of neuron differentiation (GO:0045664)	ROCK1
regulation of cytoskeleton organization (GO:0051493)	ROCK1
phosphatidylinositol biosynthetic process (GO:0006661)	CWH43
small GTPase mediated signal transduction (GO:0007264)	CCNA2
detection of chemical stimulus involved in sensory perception (GO:0050907)	OR4C6
G1/S transition of mitotic cell cycle (GO:0000082)	CCNE1
positive regulation of cellular catabolic process (GO:0031331)	ROCK1
protein phosphorylation (GO:0006468)	CCNA2;CCNE1
regulation of Wnt signaling pathway (GO:0030111)	CTNND2
regulation of cell motility (GO:2000145)	ROCK1
regulation of protein binding (GO:0043393)	ROCK1
peptidyl-lysine modification (GO:0018205)	SENP5
chromatin remodeling (GO:0006338)	KMT2B
protein localization to cell periphery (GO:1990778)	ROCK1
plasma membrane bounded cell projection organization (GO:0120036)	ROCK1
regulation of phosphorylation (GO:0042325)	FN1
positive regulation of nucleic acid-templated transcription (GO:1903508)	PRMT2;CCNE1
dephosphorylation (GO:0016311)	TPTE
platelet degranulation (GO:0002576)	FN1
protein dephosphorylation (GO:0006470)	TPTE
G2/M transition of mitotic cell cycle (GO:0000086)	CCNA2

cellular protein modification process (GO:0006464)	CCNE1;FN1;TPTE
cell cycle G2/M phase transition (GO:0044839)	CCNA2
protein lipidation (GO:0006497)	CWH43
protein localization to plasma membrane (GO:0072659)	ROCK1
muscle contraction (GO:0006936)	ROCK1
negative regulation of sequence-specific DNA binding transcription factor activity (GO:0043433)	PRMT2
neuron development (GO:0048666)	ROCK1
regulated exocytosis (GO:0045055)	FN1
protein localization to membrane (GO:0072657)	ROCK1
positive regulation of transcription, DNA-templated (GO:0045893)	PRMT2;CCNE1;KMT2B
regulation of nucleic acid-templated transcription (GO:1903506)	ZNF595;PRMT2
regulation of angiogenesis (GO:0045765)	ROCK1
regulation of cellular macromolecule biosynthetic process (GO:2000112)	ZNF595;PRMT2
protein complex assembly (GO:0006461)	FN1
positive regulation of multicellular organismal process (GO:0051240)	ROCK1
regulation of autophagy (GO:0010506)	ROCK1
regulation of MAPK cascade (GO:0043408)	FN1
regulation of canonical Wnt signaling pathway (GO:0060828)	CTNND2
positive regulation of cell migration (GO:0030335)	FN1
extracellular matrix organization (GO:0030198)	FN1
regulation of ERK1 and ERK2 cascade (GO:0070372)	FN1
protein deubiquitination (GO:0016579)	CCNA2
positive regulation of programmed cell death (GO:0043068)	PRMT2
regulation of protein phosphorylation (GO:0001932)	FN1
positive regulation of apoptotic process (GO:0043065)	PRMT2
sensory perception of chemical stimulus (GO:0007606)	OR4C6
regulation of transcription, DNA-templated (GO:0006355)	ZNF595;PRMT2;CCNE1
post-translational protein modification (GO:0043687)	FN1
phosphorylation (GO:0016310)	CCNE1
transmembrane receptor protein tyrosine kinase signaling pathway (GO:0007169)	ROCK1
protein modification by small protein conjugation (GO:0032446)	SENP5
positive regulation of cell proliferation (GO:0008284)	FN1
negative regulation of nucleic acid-templated transcription (GO:1903507)	PRMT2
cellular response to cytokine stimulus (GO:0071345)	FN1
neutrophil degranulation (GO:0043312)	ROCK1
neutrophil activation involved in immune response (GO:0002283)	ROCK1
cellular protein metabolic process (GO:0044267)	FN1
neutrophil mediated immunity (GO:0002446)	ROCK1
negative regulation of cellular macromolecule biosynthetic process (GO:2000113)	PRMT2
positive regulation of cellular process (GO:0048522)	FN1
negative regulation of gene expression (GO:0010629)	PRMT2
cytokine-mediated signaling pathway (GO:0019221)	FN1
regulation of cell proliferation (GO:0042127)	FN1
negative regulation of transcription, DNA-templated (GO:0045892)	PRMT2
regulation of apoptotic process (GO:0042981)	PRMT2
